# Medication adherence and illness perception among diabetic patients in Upper Egypt

**DOI:** 10.1186/s12902-025-01966-5

**Published:** 2025-10-02

**Authors:** Zeinab G. Abdelhamid, Doaa Mazen Abdel-Salam, Ghada A. Mohamed, Hosnia S. Abd El-Megeed

**Affiliations:** 1 Department of Public Health and Community Medicine, Faculty of Medicine, Assiut University, Assiut, Egypt; 2 Department of Internal Medicine, Faculty of Medicine, Assiut University, Assiut, Egypt

**Keywords:** Medication adherence, Type 2 diabetes, Predictors, Diabetic patients, Illness perception, Egypt

## Abstract

**Background:**

Effective diabetes management depends on medication adherence and the illness perception of patients. The primary aim of this study is to investigate medication adherence and illness perception and their correlates among diabetic patients in Upper Egypt.

**Methods:**

A cross sectional study was carried out from August 2022 to January 2023 among diabetic patients attending Diabetes Clinic in Upper Egypt. Data was collected through patient interviews. Morisky Medication Adherence Scale has been used to assess medication adherence. Illness perception was measured by using Brief Illness Perception Questionnaire. To identify the predictors of medication adherence and illness perception, logistic regression was performed using SPSS version 26. *P* value < 0.05 was considered statistically significant.

**Results:**

Out of 417 participants, 30.2% were low adherent to their diabetic medications. Predictors associated with low adherence to diabetic medications were; gender (AOR = 1.93, CI: 1.12–3.34), age (AOR = 0.40, CI: 0.19–0.87), education (AOR = 1.96, CI: 1.08–3.56), diabetes duration (AOR = 2.06, CI: 1.08–3.91), the presence of diabetes complications (AOR = 3.69, CI: 1.73–7.89), body mass index (AOR = 2.08, CI: 1.01–4.29), receiving health education in the last 6 months (AOR = 2.02, CI: 1.21–3.36), and illness perception (AOR = 6.70, CI: 3.62–12.40). A high level of illness perception was detected among 79.4% of the participants. High level of illness perception was significantly associated with residence (AOR = 2.48, CI: 1.37–4.51), presence of other comorbid conditions (AOR = 2.10, CI: 1.18–3.75), and price of medication (AOR = 2.53, CI: 1.07–5.99).

**Conclusions:**

Low adherence to diabetic medications was detected among 30.2% of the studied participants. Strategies aimed at enhancing adherence to diabetic medications should be compulsory. Furthermore, the findings of the current study recommend that illness perceptions of diabetic patients need to be improved.

**Supplementary Information:**

The online version contains supplementary material available at 10.1186/s12902-025-01966-5.

## Introduction

Diabetes mellitus (DM) is recognized as one of most persistent and rapidly growing challenges in public health. It’s a chronic metabolic disorder characterized mainly by high levels of glucose levels, associated globally with increased morbidity and mortality particularly in developing countries [1]. The World Health Organization (WHO) had predicted that by 2030, diabetes mellitus will rank the seventh cause of mortality globally [2]. Patients with type 2 DM are likely to have higher morbidity and mortality rates due to the condition’s prevalence, sneaky onset, and tardy diagnosis, particularly in resource-constrained developing nations like Africa [3]. Currently, 537 million people suffer from diabetes worldwide. Middle East and North Africa (MENA) Region have 73 million suffering from diabetes, and it is estimated that by 2045 this will rise to 135.7 million according to the International Diabetes Federation (IDF) in 2021 [4]. In 2017, Egypt was ranked eighth out of the ten nations/territories, with the highest number of individuals affected by diabetes, which ranged from 4.4 to 9.4, and is projected to be sixth in 2045 [4]. More than 77 percent of morbidities and 88 percent of fatalities in developing nations are attributable to DM [5]. Hence comes the importance of self-management in patients with type 2 DM in reducing complications from the disease and improving overall health outcomes [6]. Because failing to take prescribed medications as directed will result in ineffective treatment regimens, it is crucial to diabetic patients to adhere to their medications [6]. Medication adherence refers to how closely individuals with diabetes follow the treatment plan prescribed by their healthcare provider [7]. Adherence is defined as the degree to which a person follows established guidelines from a healthcare provider, how to use medications, diet, and lifestyle changes with scheduled doctor appointments and follow-ups [7]. It involves a proactive choice by the patient to take responsibility for their well-being and follow the prescribed treatment [8].

The average population’s adherence to long-term medicines is just 50%, and it is significantly lower in low-income countries, according to a World Health Organization assessment. An estimated 125 000 deaths are caused annually by non-adherence to medical treatment, which also causes 10% of hospital admissions and about 25% of all nursing home admissions. On the other hand, following a treatment plan is linked to improved patient outcomes and lower medical expenses [9]. Many different demographic, psychological, social, cultural, religious, condition, treatment, and health system-related factors might have an impact on non-adherence, which is a public health concern [10]. Diabetic patients who do not take their medications as prescribed are more likely to experience frequent hospital stays, lower treatment benefits, higher treatment costs, and more doctor visits. Individuals who do not adhere to their medicine have a mortality rate that is twice as high as other patients [11]. Illness perception refers to the cognitively ordered representation of a patient’s illness [12]. Perception is about everything regarding the illness such as illness identity, timeline of the disease, consequences, comorbid conditions, number of hospital admission, whether it can be controlled or not, and emotional response to being diabetic [12]. Perception about illness shapes self-management behavior. If patients have strong perception about treatment plan and regimen with personal control, it can lead to lower level of HbA1c and better health outcome [13]. Research has suggested that there is a link between medication adherence and the perception of illness [14, 15]. Rajpura and Nayak (2014) demonstrated that illness perception and positive thoughts about treatment are linked with drug adherence [14]. Chen et al. (2011) found a correlation between disease perception and medication adherence [15].

In Upper Egypt, no study has been conducted on illness perception among diabetic patients and studies on adherence to diabetic medication is deficient. As adherence level is still not improved, it is crucial to understand the potential predictors influencing poor medication adherence to ensure the implementation of interventions that promote diabetic therapy adherence. It becomes essential to determine whether patients are affected by predictors of drug non-adherence. Therefore, the current study investigates the correlates of medication adherence and illness perception among patients attending diabetic clinics at Assiut University Hospitals in Assiut, Egypt.

## Subjects and methods

### Study setting and design

A cross-sectional study was carried out at Assiut University Hospitals in Assiut district, Upper Egypt. Assiut district is the central district of Assiut Governorate, one of the Upper Egypt governorates, lying 375 km south of Cairo. Based on the latest census in 2017, the population of Assiut district was 974,993 [16]. Clinician consultations, health education, treatment distribution, and foot and vision screening are among the services provided at the diabetes clinic at Assiut University Hospitals. The study was conducted from August 2022 to January 2023.

### Study population

The target population for this study consisted of diabetic patients who sought care at diabetes clinics at Assiut University Hospitals. Inclusion of all diabetic patients who accepted to take part in the study and met the inclusion criteria.

### Sample size estimation

The sample size was calculated using two different approaches. Then, the researchers opted for the larger of the two sample sizes to enhance the generalizability and external validity of the results.

The Epi info version 7 StatCalc was used for sample size estimation, based on the following assumption: prevalence rate of non-adherence among Egyptian population (55.5%) [17], 5% precision, 95% confidence level, and design effect of 1. After the addition of 10% to guard against non-response, the estimated sample size was increased to 417.

### Inclusion criteria

Patients who met the diagnostic criteria of the 10 th revision of the International Classification of Diseases (ICD-10) [18] and who have been diagnosed with type 2 diabetes mellitus for a minimum of one year were included in the study. The age of the patient should be at least eighteen years to participate in the study. Agreement of the patients to participate in the study is very important.

### Exclusion criteria

Newly diagnosed diabetic patients, patients who are sick on the day of selection or not taking any drug for diabetes, and type 1 diabetic patients were excluded from the present study. In addition, psychiatric patients or patients using antidepressant medication that impair their cognitive capacity to think were excluded.

### Sampling technique

For selection of cases, a systematic random sampling technique was adopted in the present study. The average attendance to diabetic clinics is 40 patients per day and the authors aimed to take 10 patients per day. The first case was selected by simple random sampling technique and then every case was selected for every 4 th patient. If the patient was included before or did not fulfill the inclusion criteria, he or she will be excluded, and the next patient was included.

### Data collection tool

Data has been collected through semi-structured questionnaires which were filled in by direct interview with the participants of the study. The questionnaire was composed of four sections: The first section inquired about data on socioeconomic status such as age, educational level, gender, occupation, place of residence, and marital status. The second section of the questionnaire was about clinical data of the studied diabetic patients including BMI, type of prescribed drugs, diabetes duration, number of coexisting conditions, complications from diabetes, and adverse drug reactions. In addition, the latest HbA1c readings for the patients were obtained from their medical records. The Arabic version of The Morisky Medication Adherence Scale (MMAS-8) was the third section of the questionnaire [19]. It is still one of the most popular methods for evaluating patient adherence [19]. It consists of 8 questions with yes/no answers for questions 1 to 7 and a 5 item Likert scale for question 8 [0 = all the time, 1 = usually, 2 = sometimes, 3 = occasionally, 4 = never/rarely]. Each “no” response is rated as 1 and each “yes” response is rated as 0 except for item 5, in which each “yes” response is rated as 1 and each “no” response is rated as 0. For Item 8, the code (0-4) must be standardized by dividing the result by 4 to calculate a summated score. The MMAS-8 has a total score range of 0 to 8, where a score of 8 indicates high adherence, a score of 6-<8 indicates medium adherence, and a score of less than 6 indicates low adherence [19–21]. The Arabic version of MMAS-8 has adequate internal consistency (α = 0.70) [22]. Permission to use the scale was granted by Dr. Donald Morisky, the copyright holder of the instrument. The fourth section of the questionnaire was the Brief Illness Perception Questionnaire (B-IPQ). The B-IPQ is a nine-item tool that uses a 10-point Likert scale to assess how eight distinct features of disease are perceived [23]. The consequences (Item 1), timeframe (Item 2), personal control (Item 3), treatment management (Item 4), and identity (Item 5) are the five items that evaluate the representations of cognitive illness. Emotional representations are evaluated on two of the items: emotions (Item 8) and concern (Item 6). Item 7 is one that evaluates the understanding of the illness. An open-ended response item (item 9) asks patients to rank the three most significant causative factors contributing to their condition, which is used to assess the causal representation [23]. The sum of the scores for each item was used to determine the overall B-IPQ score. The following cut-off criteria were established for the B-IPQ total score: < 42 denotes a low experienced threat, 42–49 a moderate experienced threat, and ≥ 50 a high experienced threat [23]. A higher score denotes a more alarming and concerning perception of illnesses, whereas a lower score denotes a more benign perspective of illness [23]. With a Cronbach’s alpha of 0.717, the Arabic version of the B-IPQ has good internal consistency [24].

### Pilot study

A pilot study was conducted before starting to collect data on 10% of the sample size to determine feasibility of the study, understanding of the questionnaire, and the amount of time needed to complete each questionnaire. Then, three expert opinions were taken to check the validity of the questionnaire. No modifications to the questionnaire were needed because it was adequately explained and clear. The primary fieldwork did not include any of the patients that were part of the pilot trial.

### Statistical analysis

The SPSS version 26 was used for statistical analysis. For quantitative data, descriptive statistics were performed using the mean and standard deviation (SD), and for qualitative data, the number and percentage. To identify the important determinants of medication adherence and illness perception, logistic regression analysis was used. The association between medication adherence and illness perception was identified using Pearson correlation. Joint f test was used in regression analysis to test whether the predictors jointly have a statistically significant effect on medication adherence and illness perception. *P*-value less than 0.05 was considered statistically significant.

## Results

Table 1 describes the socio-demographic features of the participants. A total of 417 diabetic patients were included in the study. Males represent 42.4% of the participants while females represent 57.6%. The participants’ mean age was 48.82 ± 13.46 years. Most of the participants were urban residents (56.4%), married (89%), and had four or more children (57.5%). Furthermore, a significant proportion were unemployed or had unskilled occupations (78.2%). In terms of education level, 40% of the participants reported being unable to read or write.

**Table 1:** **S**ociodemographic features of the studied diabetic patients attending Diabetes Clinic in Upper Egypt

Personal data	No. (417) (%)
**Gender**	
Male	177 (42.4%)
Female	240 (57.6%)
**Age (years)**	
< 40	92 (22.1%)
40—< 50	119 (28.5%)
50—< 60	125 (30.0%)
> 60	81 (19.4%)
Mean ± ^a^SD (Range)	48.82 ± 13.46 (19.0–84.0)
**Education**	
Don’t read or write	167 (40.0%)
Primary school	48 (11.5%)
Preparatory school	54 (12.9%)
Secondary school	117 (28.1%)
Higher education/Postgraduate	31 (7.4%)
**Residence**	
Urban	235 (56.4%)
Rural	182 (43.6%)
**Marital status**	
Married	371 (89.0%)
Not married	46 (11.0%)
**Do you have any children? (*n* = 381)**	
Yes	362 (95.0%)
No	19 (5.0%)
**No. of children (*n* = 362)**	
1—3	154 (42.5%)
4 or more	208 (57.5%)
**Occupation**	
Not working/Unskilled	326 (78.2%)
Skilled/Free trades	47 (11.3%)
Clerk/Professional	44 (10.6%)

^a^SD: Standard Deviation

Table 2 demonstrates the clinical data of the studied participants. Most of the participants were overweight (41.2%) and obese (33.1%). Diabetes duration was from 5 to 10 years among 40.5% of the participants. Most of the participants were taking oral hypoglycemic drugs (58.5%).

**Table 2:** Clinical data of the studied diabetic patients attending Diabetes Clinic in Upper Egypt

	**No. (417)** (**%)**
**BMI**	
Normal	107 (25.7%)
Overweight	172 (41.2%)
Obese	138 (33.1%)
**Duration of diabetes (in years)**	
< 5	112 (26.9%)
5—< 10	169 (40.5%)
≥ 10	136 (32.6%)
**Medications**	
Insulin	117 (28.1%)
Oral hypoglycemic drugs	244 (58.5%)
Both	56 (13.4%)
**Medication side effects**	
Yes	272 (65.2%)
No	145 (34.8%)
**Price of medications**	
Affordable	89 (21.3%)
Unaffordable	246 (59.0%)
Free	82 (19.7%)
**Any other comorbid conditions?**	
Yes	254 (60.9%)
No	163 (39.1%)
**Other comorbid conditions(**^**a**^***n* = 254)**	
Hypertension	197 (77.6%)
Coronary heart disease	43 (16.9%)
Renal disease	10 (3.9%)
Liver disease	8 (3.1%)
Neurological disease	18 (7.1%)
Others	13 (5.1%)
**Complications of diabetes**	
Yes	316 (75.8%)
No	101 (24.2%)
**Measuring blood glucose regularly at home**	
Yes	284 (68.1%)
No	133 (31.9%)
**Last medical check-up**	
Less than 3 months	266 (63.8%)
More than 3 months	151 (36.2%)
**HbA1c:**	
Less than or equal 7%	89 (21.3%)
More than 7%	328 (78.7%)
**Receiving health education in the last 6 months:**	
Once or twice	219 (52.5%)
None	198 (47.5%)

^a^More than one answer had been reported

Nearly two-thirds of the participants experienced side effects from medication (65.2%) and faced financial challenges in affording it (59.0%). In addition, two thirds had other medical conditions, primarily hypertension (77.6%). Most of the participants suffered from complications related to diabetes mellitus (75.8%) and regularly monitored their blood glucose levels at home (68.1%). Table 2 also demonstrates that 63.8% of the participants had their last medical check-up less than three months ago and 52.5% received health education once or twice in the last 6 months. HbA1c levels were uncontrolled (more than 7%) among 78.7% of the participants.

**Fig. 1:**
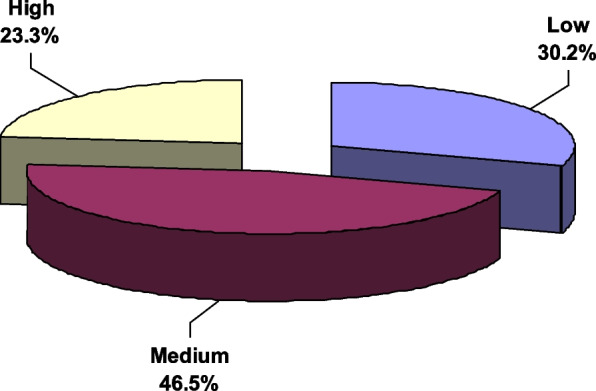
Medication Adherence among diabetic patients attending Diabetes Clinic in Upper Egypt ©*MMAS 2006* HYPERLINK "www.adherence.cc"

Figure 1 shows that 30.2% of the participants had poor adherence to their diabetes treatment. High adherence and medium adherence were reported by 23.3% and 46.5% of the participants, respectively.

**Fig. 2:**
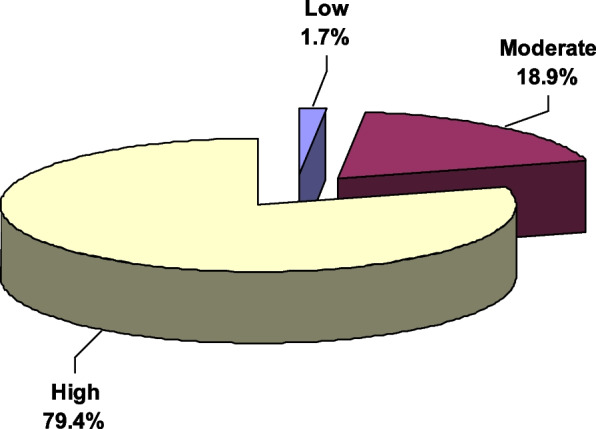
Illness Perception among diabetic patients attending Diabetes Clinic in Upper Egypt

Figure 2 demonstrates that more than two-thirds of the participants (79.4%) had a high level of illness perception. Furthermore, moderate, and low levels of illness perception were reported by 18.9% and 1.7% of the participants, respectively.

Table 3 revealed that low adherence was significantly associated with gender, age, education, duration of disease, presence of complication, body mass index (BMI), health education and illness perception in the logistic regression analysis. Concerning sex, being a female gender was positively associated with low adherence to diabetic medication (AOR = 1.93, CI: 1.12–3.34, *P* = 0.019). Furthermore, secondary school or higher education was positively associated with low adherence (AOR = 1.96, CI: 1.08–3.56, *P* = 0.027). Compared to patients with diabetes duration ≥ 10 years, those with diabetes duration ranging from 5 to 10 years were more likely to be low adherent (AOR = 2.06, CI: 1.08–3.91, *P* = 0.028). The presence of diabetes complications reduced the likelihood of adherence to diabetic therapy (AOR = 3.69, CI: 1.73–7.89, *P* = 0.001). Regarding (BMI), obese patients were positively associated with low adherence (AOR = 2.08, CI: 1.01–4.29, *P* = 0.049). Patients who didn’t receive health education in the last 6 months were more likely to be low adherent to diabetic regimen (AOR = 2.02, CI: 1.21–3.36, *P* = 0.007). Patients with low levels of illness perception were more likely to be low adherent to their medications (AOR = 6.70, CI: 3.62–12.40, *P* = 0.000). On the other hand, patients aged from 40 to less than 50 years were less likely to be low adherent to their medication (AOR = 0.40, CI: 0.19–0.87, *P* = 0.020).

**Table 3:** Logistic regression analysis of predictors of low adherence among diabetic patients attending Diabetes Clinic in Upper Egypt

	**Adherence**		**COR (95% CI)**	***P*-value**	**AOR (95% CI)**	***P*-value**
	**Medium/High**	**Low**				
	**No. (%)**	**No. (%)**				
**Gender:**						
Male (r)	137 (77.4)	40 (22.6)	–		–	
Female	154 (64.2)	86 (35.8)	1.91 (1.23–2.97)	0.004*	1.93(1.12–3.34)	0.019*
**Age: (years)**						
> 60 (r)	57 (70.4)	24 (29.6)	–		–	
50—< 60	85 (68.0)	40 (32.0)	1.12 (0.61–2.05)	0.108	1.00(0.40–2.50)	0.997
40—< 50	92 (77.3)	27 (22.7)	0.70 (0.37–1.32)		0.40(0.19–0.87)	0.020*
< 40	57 (62.0)	35 (38.0)	1.46 (0.77–2.75)		0.79(0.38–1.65)	0.538
**Residence:**						
Rural (r)	139 (76.4)	43 (23.6)	–		–	
Urban	152 (64.7)	83 (35.3)	1.77 (1.14–2.72)	0.010*	1.10(0.64–1.897)	0.723
**Education:**						
Doesn’t read or write/basic education (r)	196 (72.9)	73 (27.1)	–		–	
Secondary school/High education	95 (64.2)	53 (35.8)	1.50 (0.97–2.30)	0.065	1.96(1.08–3.56)	0.027*
**Marital status:**						
Married (r)	259 (69.8)	112 (30.2)	–		–	
Not married	32 (69.6)	14 (30.4)	1.01 (0.52–1.97)	0.973	1.17(0.49–2.81)	0.724
**Duration of disease: (years)**						
≥ 10 (r)	102 (75.0)	34 (25.0)	–		–	
5—< 10	125 (74.0)	44 (26.0)	1.06 (0.63–1.77)	0.003*	2.06(1.08–3.91)	0.028*
< 5	64 (57.1)	48 (42.9)	2.25 (1.31–3.86)		1.06(0.58–1.92)	0.861
**Medication side effects:**						
Yes (r)	192 (70.6)	80 (29.4)	–		–	
No	99 (68.3)	46 (31.7)	1.12 (0.72–1.73)	0.624	1.13(0.67–1.90)	0.635
**Any medical conditions:**						
No (r)	116 (71.2)	47 (28.8)	–		–	
Yes	175 (68.9)	79 (31.1)	1.11 (0.72–1.72)	0.623	1.11(0.62–2.00)	0.726
**Complications of DM:**						
No (r)	83 (82.2)	18 (17.8)	–		–	
Yes	208 (65.8)	108 (34.2)	2.39 (1.37–4.19)	0.002*	3.69(1.73–7.89)	0.001*
**Blood glucose measure:**						
Yes (r)	200 (70.4)	84 (29.6)	–		–	
No	91 (68.4)	42 (31.6)	1.10 (0.70–1.72)	0.678	1.14(0.66–1.98)	0.635
**Last medical check-up:**						
Less than 3 months (r)	187 (70.3)	79 (29.7)	–		–	
More than 3 months	104 (68.9)	47 (31.1)	1.07 (0.69–1.65)	0.760	1.177(0.69–2.02)	0.555
**HbA1c:**						
Less than or equal 7% (r)	62 (69.7)	27 (30.3)	–		–	
More than 7%	229 (69.8)	99 (30.2)	0.99 (0.60–1.65)	0.978	0.77(0.41–1.47)	0.432
**BMI:**						
Normal (r)	80 (74.8)	27 (25.20)	–		–	
Overweight	119 (69.2)	53 (30.8)	1.32 (0.77–2.27)	0.382	1.80(0.93–3.49)	0.083
Obese	92 (66.7)	46 (33.3)	1.48 (0.84–2.60)		2.08(1.01–4.29)	0.049*
**Price of medications:**						
Affordable (r)	69 (77.5)	20 (22.5)	–		–	
Unbearable	168 (68.3)	78 (31.7)	1.60 (0.91–2.82)	0.183	1.35(0.69–2.65)	0.386
Free	54 (65.9)	28 (34.1)	1.79 (0.91–3.51)		1.76(0.77–4.03)	0.179
**Health education last 6 m:**						
Once or twice (r)	163 (74.4)	56 (25.6)	–		–	
None	128 (64.6)	70 (35.4)	1.59 (1.05–2.43)	0.030*	2.02(1.21–3.36)	0.007*
**Medication:**						
Single (r)	252 (69.8)	109 (30.2)	–		–	
Multiple	39 (69.6)	17 (30.4)	1.01 (0.55–1.86)	0.980	0.95(0.46–1.99)	0.897
**Illness perception** High (r)	254 (76.7)	77 (23.3)	–		–	0.000*
Low/medium	37 (43)	49 (57)	4.37 (2.66–7.18)	0.000*	6.70(3.62–12.40)	

Table 4 showed that the joint effect of the predictors of medication adherence was statistically significant (f = 6.595, *P* = 0.000).

**Table 4:** Joint F test for overall contribution of predictors of medication adherence

	**Sum of Squares**	**df**	**Mean Square**	**F**	**Sig**
Regression	363.001	17	21.353	6.595	0.000
Residual	1291.885	399	3.238		
Total	1654.886	416			

Table 5 depicted that high level of illness perception was significantly associated with residence, presence of other comorbid conditions, and price of medication in the logistic regression analysis. Rural patients experienced a high level of illness perception compared to urban residents (AOR = 2.48, CI: 1.37–4.51, P = 0.003). Furthermore, patients with other medical conditions had a higher level of illness perception (AOR = 2.10, CI: 1.18–3.75, *P* = 0.012). Patients who found the cost of medication affordable showed an increased level of illness perception when compared to those receiving medication for free (AOR = 2.53, CI: 1.07–5.99, *P* = 0.035).

**Table 5:** Logistic regression analysis of predictors of high level of illness perception among diabetic patients attending Diabetes Clinic in Upper Egypt

	**Illness Perception**		**COR (95% CI)**	***P*-value**	**AOR (95% CI)**	***P*-value**
	**Low/moderate**	**High**				
	**No. (%)**	**No. (%)**				
**Gender:**						
Male (r)	39 (22.0)	138 (78.0)			–	
Female	47 (19.6)	193 (80.4)	1.16 (0.72–1.87)	0.541	1.01 (0.57–1.79)	0.970
**Age: (years)**						
< 40 (r)	24 (26.1)	68 (73.9)	–		–	
40—< 50	23 (19.3)	96 (80.7)	1.47 (0.77–2.82)	0.475	0.98 (0.44–2.17)	0.950
50—< 60	22 (17.6)	103 (82.4)	1.65 (0.86–3.18)		0.86 (0.37–1.97)	0.718
> 60	17 (21.0)	64 (79.0)	1.33 (0.65–2.70)		0.75 (0.30–1.88)	0.539
**Residence:**						
Urban (r)	56 (23.8)	179 (76.2)	–		–	
Rural	30 (16.5)	152 (83.5)	1.59 (0.97–2.60)	0.066	2.48 (1.37–4.51)	0.003*
**Education:**						
Secondary school/ High education (r)	33 (22.3)	115 (77.7)	–		–	
Doesn’t read or write/ basic education	53 (19.7)	216 (80.3)	1.17 (0.72–1.91)	0.531	0.99 (0.53–1.84)	0.969
**Duration of disease: (years)**						
< 5 (r)	26 (23.2)	86 (76.8)	–		–	
5—< 10	30 (17.8)	139 (82.2)	1.40 (0.78–2.53)	0.477	1.33 (0.69–2.56)	0.391
≥ 10	30 (22.1)	106 (77.9)	1.07 (0.59–1.94)		0.92 (0.47–1.80)	0.806
**Medication side effects:**						
No (r)	36 (24.8)	109 (75.2)	–		–	
Yes	50 (18.4)	222 (81.6)	1.47 (0.90–2.38)	0.121	1.41 (0.83–2.41)	0.207
**Any medical conditions:**						
No (r)	47 (28.8)	116 (71.2)	–		–	
Yes	39 (15.4)	215 (84.6)	2.23 (1.38–3.61)	0.001*	2.10 (1.18–3.75)	0.012*
**Complications of DM:**						
No (r)	29 (28.7)	72 (71.3)	–		–	
Yes	57 (18.0)	259 (82.0)	1.83 (1.09–3.07)	0.021*	1.75 (0.91–3.38)	0.092
**Blood glucose measure:**						
Yes (r)	61 (21.5)	223 (78.5)	–		–	
No	25 (18.8)	108 (81.2)	1.18 (0.70–1.99)	0.528	1.01 (0.56–1.82)	0.968
**Last medical check-up:**						
Less than 3 months (r)	58 (21.8)	208 (78.2)	–		–	
More than 3 months	28 (18.5)	123 (81.5)	1.22 (0.74–2.03)	0.429	1.36 (0.77–2.42)	0.290
**HbA1c:**						
More than 7% (r)	69 (21.0)	259 (79.0)	–		–	
Less than or equal 7%	17 (19.1)	72 (80.9)	1.13 (0.62–2.04)	0.689	1.38 (0.69–2.74)	0.361
**BMI:**						
Normal (r)	31 (29.0)	76 (71.0)	–		–	
Overweight	30 (17.4)	142 (82.6)	1.93 (1.09–3.43)	0.046*	1.49 (0.78–2.85)	0.223
Obese	25 (18.1)	113 (81.9)	1.84 (1.01–3.37)		1.44 (0.72–2.88)	0.299
**Price of medications:**						
Free (r)	23 (28.0)	59 (72.0)	–		–	
Affordable	15 (16.9)	74 (83.1)	1.92 (0.92–4.01)	0.156	2.53 (1.07–5.99)	0.035*
Unbearable	48 (19.5)	198 (80.5)	1.61 (0.90–2.86)		1.62 (0.85–3.09)	0.142
**Health education last 6 m:**						
None (r)	43 (21.7)	155 (78.3)	–		–	
Once or twice	43 (19.6)	176 (80.4)	1.14 (0.71–1.83)	0.600	1.10 (0.65–1.85)	0.725
**Medication:**						
Single (r)	80 (22.2)	281 (77.8)	–		–	
Multiple	6 (10.7)	50 (89.3)	2.37 (0.98–5.73)	0.049*	2.19 (0.85–5.65)	0.106

Table 6 revealed that the joint effect of the predictors of illness perception was statistically significant (f = 3.247, *P* = 0.000).

**Table 6:** Joint F test for overall contribution of predictors of illness perception

	**Sum of Squares**	**df**	**Mean Square**	**F**	**Sig**
Regression	1877.051	16	117.316	3.247	0.000
Residual	14451.947	400	36.130		
Total	16328.998	416			

**Fig. 3:**
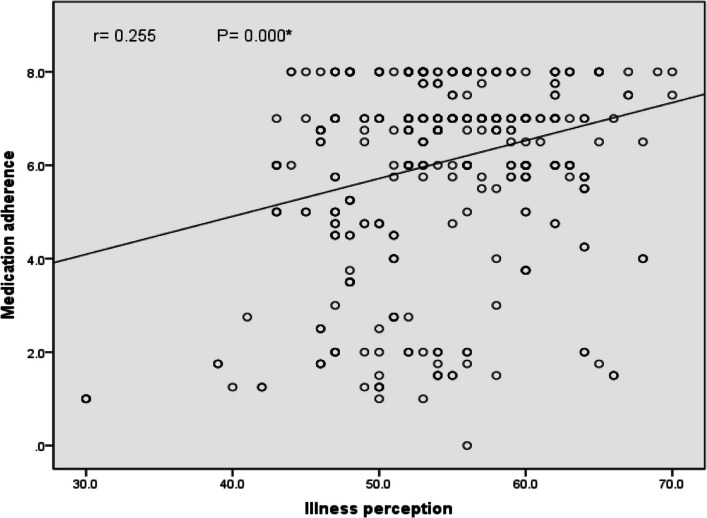
Correlation between illness perception and medication adherence among diabetic patients attending Diabetes Clinic in Upper Egypt

Figure 3 showed a significant positive correlation between level of illness perception and medication adherence, indicating that high level of illness perception was associated with better adherence to medications (r = 0.255, *P* = 0.000).

## Discussion

Diabetes mellitus represents a significant global public health concern. The World Health Organization has released various reports that emphasize the grave repercussions of non-compliance with therapy among individuals with diabetes. Consequently, it is imperative to prioritize efforts aimed at promoting adherence to therapy over the development of novel medical interventions. This is because, irrespective of the level of innovation achieved in treatment modalities, their effectiveness will be nullified if patients fail to adhere to them [25]. Therefore, the present study determines the degree of adherence with diabetes medication and its associated factors.

Approximately 30.2% of the patients in this study were poor adherent to their diabetic medications. The finding of the present study was concordant with other studies conducted by Waari et al. and Gelaw et al. who found that 28.3% and 28% of the patients had low medication adherence, respectively [26, 27]. A study conducted by Heissam et al. in Egypt revealed that 26% of the participants had poor adherence to diabetic medications [28]. A French population-based study revealed that only 12% of the participants had poor adherence to diabetic medications [29]. Concerning Gulf countries, the rate of poor medication adherence in this study is higher than that revealed by Aloudah et al. and Alqarni et al. (23% and 21.4%, respectively) [30, 31]. On the other hand, poor adherence in this study is lower than that reported by a Sudanese study which showed that 40.4% of the studied diabetic patients were low adherent to their therapy [32].

The review conducted by Krass et al. includes 27 studies with medication adherence rates ranging from 38.5% to 93.1%. Out of 27 studies, only six found a prevalence of adherence equal to or more than 80% [33]. Comparing adherence rates among patients with type 2 diabetes mellitus across different countries or even within different regions within the same country proves challenging attitude due to variations in lifestyle factors, policies and strategies implemented by different nations. Furthermore, differences in awareness regarding medication adherence importance, utilization of diverse measurement tools for adherence assessment, and reliance on patient self-reporting, which may not always be entirely accurate are other possible causes for the variability in adherence rate.

In developing counties, females are responsible for pursuance of daily activities in their families and this huge burden could predispose them to be less adherent to their medication. The present study showed that females were more likely to be less adherent to diabetic medications compared to males. This association agreed with the other studies [34, 35]. Participants with high educational level were less adherent to their diabetic medications in the present study in contradiction with other studies [36, 37]. This may be explained by the fact that highly educated personnel have less trust in medical care [38].

In line with a study done by Marinho et al., this study demonstrated that younger age was independently related to better adherence to medications [39]. In addition, this study revealed that diabetic patients with diabetes duration of 5 to 10 year were more likely to be less adherent to their diabetic medications in contradiction with other studies [39, 40].

A possible explanation might be that old people or those with diabetes for a duration of 5 to 10 years are adapted to their disease with subsequent less contact with a physician or diabetes educator, which can lead to less medication adherence.

As the challenges associated with obesity, such as reduced physical activity, bad dietary habits, and potential comorbidities, can complicate the management of diabetes. Additionally, obesity is often linked with psychological aspects, including lower health literacy, stigma, and mental health problems, which may affect an individual’s ability to adhere to a structured treatment plan. In this study, obese patients were prone to have low medication adherence, in agreement with other studies [41–43]

Patients with low level of illness perception exhibit low adherence to treatment as the present study showed. Patients with low level of illness perception underestimate the severity of the condition; they might downplay the importance of adherence to prescribed treatments and lifestyle modifications. This perception could stem from a lack of awareness about the potential complications of diabetes. Conversely, patients with high levels of illness perception may be more aware of the potential consequences, motivating them to adhere more to treatment plans, this agrees with multiple studies [44–46].

According to this study, patients with complications from diabetes had a higher likelihood of being less adherent to medications. This could be explained by the fact that individuals are less likely to think that diabetic drugs are effective after they experience difficulties caused by diabetes. This result was in line with previous research [35, 47], which indicated that diabetic complications were a contributing factor for low adherence among diabetic patients.

Patient adherence to treatment is greatly influenced by their understanding of the disease process, potential side effects and complications, as well as the principles of therapy, which can be acquired through health education. This study showed that diabetic patients who didn’t receive any health education sessions were more likely to have low adherence to their medications. This finding was supported by other studies [48, 49], highlighting the importance of providing diabetes clinics with knowledgeable and well-trained healthcare professionals. By doing so, patients can gain a better understanding of their condition and improve their adherence to therapy. Illness perception is defined as patient’s cognitive evaluation and personal comprehension of a medical condition and its possible outcomes [50]. Concerning the predictors of illness perception, some factors such as rural residence, presence of other comorbid conditions, and an affordable cost of medications contributed significantly to high level of illness perception in the present study. A unique observation in this study is rural residents experienced high level of illness perception despite their low level of education and low access to healthcare information. This high level of illness perception makes them more adherent to their medications compared to urban residents as shown in this study. Patients with other comorbidity conditions encountered more serious symptoms and believed that this illness condition had a serious impact on their lives. In this study, patients with other comorbid conditions have high level of illness perception in agreement with a study conducted by Murugesan & Sundaramoorthy [51]. The present study was conducted in governmental hospitals, where most patients ask for free medications. When patients observe that the government ensures that the medications are affordable and available, it raises their awareness of diabetes as a significant public health problem. This study has shed light on the role of the cost of medications in shaping illness perception among diabetic patients. It showed that patients who found the price of their medication affordable tended to have a high level of illness perception. The affordability of medication may influence patients’ perceptions of the severity of their condition and the importance of the disease [52]. The present study not only contributes to the understanding of how patients perceive diabetes, but it also brings to the forefront three distinct factors such as residence, co-morbid conditions, and medication affordability, that are often overlooked in the existing body of research. This unique aspect of this study not only reinforces its significance but also highlights its potential to influence policy and healthcare initiatives, especially in regions such as Upper Egypt, where these factors have been historically underrepresented and where our findings could serve as a catalyst for targeted interventions and improvements in healthcare delivery. The present study revealed that medication adherence was associated significantly with illness perception. This finding agreed with studies conducted in Iran by Pour et al. and Bilondi et al. which showed that lack of illness perception is one of the crucial factors linked with medication non-adherence [53, 54].

The limitation of the present study is that it was conducted at a single center, whereas a multicenter study would have increased the findings’ generalizability. Another limitation of the present study was using an interview questionnaire with subsequent recall bias and exaggerated response among the participants.

## Conclusions

The findings of the present study showed that the rate of non-adherence to medications among the participants was considerably high. Medication nonadherence was significantly associated with gender, age, education, diabetes duration, the presence of diabetes complications, body mass index, receiving health education, and illness perception. Determining the level of medication adherence and predictors that indicate optimal adherence in patients with diabetes is essential to enable stakeholders to take all necessary initiatives to address these issues. The present study revealed that more than two-thirds of the participants had a high level of illness perception. High level of illness perception was significantly associated with residence, presence of other comorbid conditions, and price of medication. These findings could improve our understanding of illness perceptions and help us to develop interventions that improve patients’ perceptions of their illnesses.

## Supplementary Information

Supplementary Material 1.

Supplementary Material 2.

Supplementary Material 3.

Supplementary Material 4.

## Data Availability

No datasets were generated or analysed during the current study.
