# Correction: Medication adherence and illness perception among diabetic patients in Upper Egypt

**DOI:** 10.1186/s12902-026-02264-4

**Published:** 2026-06-05

**Authors:** Zeinab G. Abdelhamid, Doaa Mazen Abdel‑Salam, Ghada A. Mohamed, Hosnia S. Abd El‑Megeed

**Affiliations:** 1 Department of Public Health and Community Medicine, Faculty of Medicine, Assiut University, Assiut, Egypt; 2 Department of Internal Medicine, Faculty of Medicine, Assiut University, Assiut, Egypt

**Correction: BMC Endocr Disord (2025) 25:223**

10.1186/s12902-025-01966-5

Following publication of the original article [1], the corrections below have been made to ensure accurate description and attribution of the MMAS 8 instrument publication review.

1. In the ‘‘Subjects and Methods’’ section under the subheading “Data collection tool” subsection, the 7th sentence:
  The MMAS-8 has a total score range of 0 to 8, where a score of 8 indicates high adherence, a score of 7 or 6 indicates medium adherence, and a score of less than 6 indicates low adherence [19]. The Arabic version of MMAS-8 has adequate internal consistency (α = 0.70) [20].
  Has been replaced with:
  Each ‘‘no’’ response is rated as 1 and each ‘‘yes’’ response is rated as 0 except for item 5, in which each ‘‘yes’’ response is rated as 1 and each ‘‘no’’ response is rated as 0. For Item 8, the code (0–4) must be standardized by dividing the result by 4 to calculate a summated score. The MMAS-8 has a total score range of 0 to 8, where a score of 8 indicates high adherence, a score of 6-<8 indicates medium adherence, and a score of less than 6 indicates low adherence [19–21]. The Arabic version of MMAS-8 has adequate internal consistency (α = 0.70) [22]. Permission to use the scale was granted by Dr. Donald Morisky, the copyright holder of the instrument.
2. Two new references [2, 3] have been added accordingly in the original article as:
  [20] Berlowitz DR, Foy CG, Kazis LE, Bolin L, Lonroy LB, Fitzpatrick P, et al. for the SPRINT Study Research Group. Impact of Intensive Blood Pressure Therapy on Patient-Reported Outcomes: Outcomes Results from the SPRINT Study. N Engl J Med. 2017; 377:733–744.
  [21] Bress AP, Bellows BK, King J, Hess R Beddhu S, Zhang Z, et al. for the SPRINT Research Group and the SPRINT Economics and Health Related Quality of Life Subcommittee. Cost- Effectiveness of Intensive versus Standard Blood Pressure Control. N Engl J Med. 2017; 377:745–755.
3. The caption of Fig. 1 has been updated from:
  **Fig. 1** Medication Adherence among diabetic patients attending Diabetes Clinic in Upper Egypt.
  To:
  **Fig. 1** Medication Adherence among diabetic patients attending Diabetes Clinic in Upper Egypt *©MMAS 2006* www.adherence.cc.
4. The following sentence has been added to the end of acknowledgement section: “The authors would like to sincerely thank Dr. Donald Morisky for granting permission to use the Morisky Medication Adherence Scale (MMAS) in this study. *©MMAS 2006* www.adherence.cc*”*.

The original article [1] has been updated.
