# Supplementary material for: Medication adherence and illness perception among diabetic patients in Upper Egypt
Source: BMC Endocr Disord. 2025 Oct 2;25:223. doi: 10.1186/s12902-025-01966-5 (PMC12492867; doi:10.1186/s12902-025-01966-5)
Supplement: Supplementary file 1 — Supplementary Material 1. [file 12902_2025_1966_MOESM1_ESM.docx]

|  | Sum of Squares | df | Mean Square | F | Sig. |
| --- | --- | --- | --- | --- | --- |
| Regression | 363.001 | 17 | 21.353 | 6.595 | .000 |
| Residual | 1291.885 | 399 | 3.238 |  |  |
| Total | 1654.886 | 416 |  |  |  |

**Table 1: Joint F test for predictors of medication adherence**

This table indicates that the regression model is statistically significant, with an F-value of 6.595 and a p-value of 0.000.

| Model | (B) | Std. Error | (Beta) | t | Sig. | 95.0% Confidence Interval for B |
| --- | --- | --- | --- | --- | --- | --- |
| Constant | 4.85 | 1.15 |  | 4.22 | 0.00 | (2.59, 7.11) |
| Gender | -0.36 | 0.20 | -0.09 | -1.83 | 0.07 | (-0.75, 0.03) |
| Age (years) | 0.03 | 0.10 | 0.02 | 0.32 | 0.75 | (-0.17, 0.23) |
| Education | -0.62 | 0.23 | -0.15 | -2.70 | **0.01*** | (-1.08, -0.17) |
| Residence | 0.63 | 0.20 | 0.16 | 3.24 | **0.00*** | (0.25, 1.02) |
| Marital status | -0.05 | 0.31 | -0.01 | -0.16 | 0.87 | (-0.66, 0.56) |
| Occupation | 0.13 | 0.16 | 0.04 | 0.79 | 0.43 | (-0.19, 0.44) |
| BMI | -0.27 | 0.13 | -0.10 | -2.11 | **0.04*** | (-0.51, -0.02) |
| Duration of disease (years) | 0.19 | 0.12 | 0.08 | 1.58 | 0.12 | (-0.05, 0.44) |
| Type of medications | 0.10 | 0.28 | 0.02 | 0.35 | 0.73 | (-0.45, 0.64) |
| Price of medications | -0.01 | 0.15 | -0.00 | -0.06 | 0.95 | (-0.31, 0.29) |
| Any medical conditions | 0.18 | 0.21 | 0.05 | 0.87 | 0.39 | (-0.23, 0.60) |
| Complications of DM | 0.68 | 0.25 | 0.15 | 2.69 | **0.01*** | (0.18, 1.17) |
| Blood glucose measure | -0.25 | 0.20 | -0.06 | -1.26 | 0.21 | (-0.65, 0.14) |
| Last medical check-up | 0.21 | 0.20 | 0.05 | 1.08 | 0.28 | (-0.17, 0.60) |
| HbA1c | -0.57 | 0.23 | -0.12 | -2.42 | **0.02*** | (-1.03, -0.11) |
| Health education last 6 months | -0.54 | 0.18 | -0.12 | -2.93 | **0.00*** | (-0.89, -0.18) |
| Perception (B-IPQ level) | 1.31 | 0.23 | 0.27 | 5.73 | **0.00*** | (0.86, 1.76) |

This table shows that several variables were significantly associated with medication adherence such as residence, education, BMI, complications of DM, HbA1c, health education, and illness perception.

**Table 2: Joint F test for predictors of illness perception**

|  | Sum of Squares | df | Mean Square | F | Sig. |
| --- | --- | --- | --- | --- | --- |
| Regression | 1877.051 | 16 | 117.316 | 3.247 | **0.000*** |
| Residual | 14451.947 | 400 | 36.130 |  |  |
| Total | 16328.998 | 416 |  |  |  |

This table indicates that the regression model is statistically significant, with an F-value of 3.247 and a p-value of 0.000, indicating that the regression model is statistically significant.

| Model | (B) | Std. Error | (Beta) | t | Sig. | 95.0% Confidence Interval for B |
| --- | --- | --- | --- | --- | --- | --- |
| (Constant) | 50.71 | 3.59 |  | 14.12 | 0.00* | (43.65, 57.78) |
| Gender | 1.54 | 0.66 | 0.12 | 2.33 | **0.02*** | (0.24, 2.84) |
| Age (years) | -0.08 | 0.34 | -0.01 | -0.23 | 0.82 | (-0.74, 0.58) |
| Education | 0.77 | 0.77 | 0.06 | 1.00 | 0.32 | (-0.75, 2.29) |
| Residence | 1.18 | 0.65 | 0.09 | 1.82 | **0.07*** | (-0.10, 2.45) |
| Marital status | 0.78 | 1.03 | 0.04 | 0.76 | 0.45 | (-1.24, 2.81) |
| Occupation | -0.68 | 0.53 | -0.07 | -1.29 | 0.20 | (-1.73, 0.36) |
| BMI | 0.23 | 0.42 | 0.03 | 0.56 | 0.58 | (-0.59, 1.06) |
| Duration of disease (years) | 0.52 | 0.41 | 0.06 | 1.27 | 0.21 | (-0.29, 1.33) |
| Type of medications | 1.91 | 0.92 | 0.10 | 2.08 | **0.04*** | (0.11, 3.72) |
| Price of medications | -0.01 | 0.50 | -0.00 | -0.02 | 0.99 | (-0.99, 0.98) |
| Any medical conditions | -2.16 | 0.70 | -0.17 | -3.09 | **0.00*** | (-3.53, -0.79) |
| Complications of DM | -1.92 | 0.84 | -0.13 | -2.29 | **0.02*** | (-3.57, -0.28) |
| Blood glucose measure | 0.47 | 0.67 | 0.04 | 0.69 | 0.49 | (-0.86, 1.79) |
| Last medical check-up | 0.46 | 0.65 | 0.04 | 0.71 | 0.48 | (-0.82, 1.74) |
| HbA1c | -0.95 | 0.78 | -0.06 | -1.21 | 0.23 | (-2.48, 0.59) |
| Health education last 6 months | 0.56 | 0.61 | 0.05 | 0.92 | 0.36 | (-0.64, 1.76) |

The table shows that several variables were significantly associated with illness perception such as gender, residence, type of medications, the presence of medical conditions, and the presence of complications of diabetes mellitus (DM)
