# Supplementary figures and images for: Medication adherence and illness perception among diabetic patients in Upper Egypt
Source: BMC Endocr Disord. 2025 Oct 2;25:223. doi: 10.1186/s12902-025-01966-5 (PMC12492867; doi:10.1186/s12902-025-01966-5)

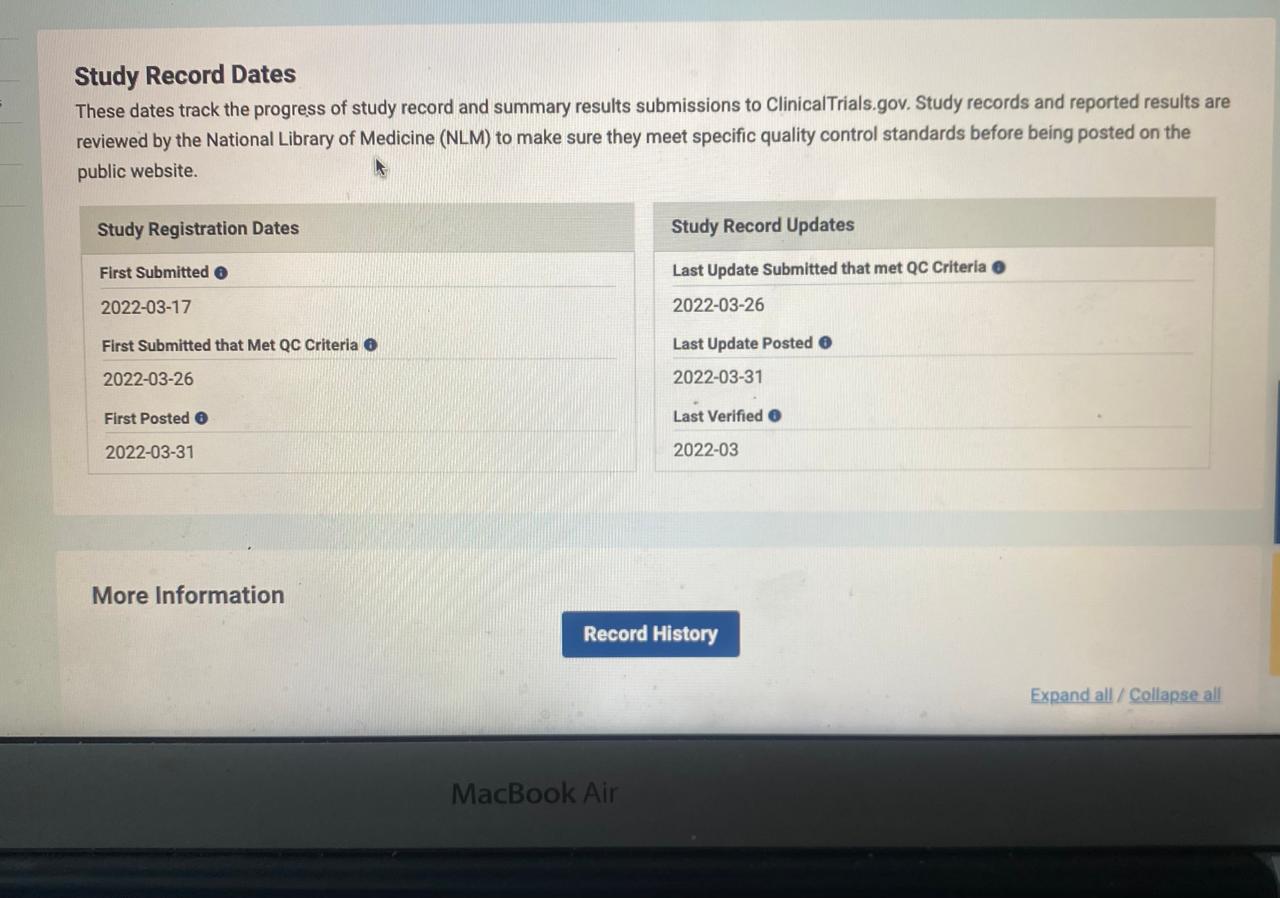

Supplement: Supplementary file 2 — Supplementary Material 2. [file 12902_2025_1966_MOESM2_ESM.jpeg]
