# Supplementary material for: Medication adherence and illness perception among diabetic patients in Upper Egypt
Source: BMC Endocr Disord. 2025 Oct 2;25:223. doi: 10.1186/s12902-025-01966-5 (PMC12492867; doi:10.1186/s12902-025-01966-5)
Supplement: Supplementary file 3 — Supplementary Material 3. [file 12902_2025_1966_MOESM3_ESM.pdf]

20-Aug-2024

Certificate Number: 9968-9833-5849-8846-6423

To Whom It May Concern:

This is to inform you that Zeinab Galal Eldeen Abdelhamid, Demonstrator of Public Health & Community Medicine, Faculty of Medicine, Assiut University has my permission to use the MMAS-8 (Morisky Medication Adherence Scale 8 item U.S. Reg. No. TX-8-632-533) in this research study and for publication in all formats i.e. print and digital:

Adherence, Beliefs about Medications, and Illness Perception among Type 2 Diabetic Patients in Assiut University Hospital "

The requirements to use this scale are to cite the following references in the document:

1. **Berlowitz DR, Foy CG, Kazis LE, Bolin L, Lonroy LB, Fitzpatrick P, et al. for the SPRINT Study Research Group. Impact of Intensive Blood Pressure Therapy on Patient-Reported Outcomes: Outcomes Results from the SPRINT Study. N Engl J Med 2017; 377:733-44.**

2. **Bress AP, Bellows BK, King J, Hess R Beddhu S, Zhang Z, et al, for the SPRINT Research Group and the SPRINT Economics and Health Related Quality of Life Subcommittee. Cost- Effectiveness of Intensive versus Standard Blood Pressure Control. N Engl J Med 2017; 377:745-55.**

Terms and conditions are found here:
https://adherence.cc/mmas-8

i. The following footnote is required in all articles, presentations, web postings, reports and submitted manuscripts, and on the first table or figure which present the MMAS-8 as well as in the Acknowledgment Section of manuscripts submitted for publication:

©MMAS 2006 www.adherence.cc

Donald E. Morisky

Donald E. Morisky, Sc.D., M.S.P.H., Sc.M. President

Philip Morisky, MBA
Chief Optimus
adherence.
www.adherence.cc
20-Aug-2024

Researcher permission letter_Zeinab Galal Eldeen Abdelhamid

Final Audit Report 2024-08-20

Created:	2024-08-20
By:	Philip Morisky (philm715@icloud.com)
Status:	Signed
Transaction ID:	CBJCHBCAABAAOM0Aew8IuCUdujpJNsw9nGoJCxllQ6SI

"Researcher permission letter_Zeinab Galal Eldeen Abdelhamid" History

Document created by Philip Morisky (philm715@icloud.com)
2024-08-20 - 10:22:13 PM GMT- IP address: 66.214.146.130

Document emailed to Philip Morisky (philip.morisky@adherence.cc) for signature
2024-08-20 - 10:22:16 PM GMT

Email viewed by Philip Morisky (philip.morisky@adherence.cc)
2024-08-20 - 10:22:33 PM GMT- IP address: 66.214.146.130

Document e-signed by Philip Morisky (philip.morisky@adherence.cc)
Signature Date: 2024-08-20 - 10:23:02 PM GMT - Time Source: server- IP address: 66.214.146.130

Agreement completed.
2024-08-20 - 10:23:02 PM GMT

Adobe Acrobat Sign
