# Supplementary material for: Medication adherence and illness perception among diabetic patients in Upper Egypt
Source: BMC Endocr Disord. 2025 Oct 2;25:223. doi: 10.1186/s12902-025-01966-5 (PMC12492867; doi:10.1186/s12902-025-01966-5)
Supplement: Supplementary file 4 — Supplementary Material 4. [file 12902_2025_1966_MOESM4_ESM.pdf]

Questionnaire on Medication Adherence and Illness Perception among Diabetic Patients in Upper Egypt

**Sociodemographic data:**		
1	Gender	1) Male 2) Female
2	Age	
3	Education	1) Doesn’t read or write<br>2) Primary education<br>3) preparatory education<br>4) Secondary education<br>5) University<br>6) Post graduate education
4	Residence	1) Urban<br>2) rural
5	Marital status	1) Married<br>2) Single (move to Q8)<br>3) Divorced<br>4) Widowed
6	Do you have any children?	1) Yes<br>2) No
7	If yes, how many?	
8	Occupation	1) Unemployed<br>2) Unskilled worker<br>3) Skilled worker<br>4) Unprofessional Job<br>5) Professional Job
**Clinical data**		
9	Height	
10	Weight	
11	How long since do you have diabetes mellitus type 2?	
12	Type and number of antihyperglycemic drugs prescribed by your doctor:	1) Insulin<br>2) Oral hypoglycemia medications<br>3) Both

**13**	**did you have any side effects from your prescribed medications?**	**1) Yes**<br>**2) No**
**14**	**Is the price of medicines suitable for you?**	**1) Reasonable**<br>**2) Unbearable**<br>**3) Free**
**15**	**Do you suffer from any other medical conditions?**	**1) Yes**<br>**2) No**
**16**	**So yes, what are these conditions?**	
**17**	**Do you suffer from any complications due to diabetes?**	**1) Yes**<br>**2) No**
**18**	**Do you measure your blood glucose level at home?**	**1) Yes**<br>**2) No**
**19**	**What was your last medical examination?**	**1) 3 months ago**<br>**2) More than 3 months**
**20**	**What is the latest percentage of HbA1c for patients from medical records?**	**1) Controlled (less than or equal to 7%)**<br>**2) Uncontrolled (more than 7%)**
**21**	**Have you received diabetes education in the last 6 months?**	**1) Once or twice**<br>**2) Not even once**
**Morisky Medication Adherence Scale (MMAS)**		
**22**	**Do you sometimes forget to take your medications?**	**1) Yes**<br>**2) No**
**23**	**People sometimes miss taking their medications for reasons other than forgetting. Thinking over the past two weeks, were there any days when you did not take your medications?**	**1) Yes**<br>**2) No**
**24**	**Have you ever cut back or stopped taking your medications without telling your doctor, because you felt worse when you took it?**	**1) Yes**<br>**2) No**
**25**	**When you travel or leave home, do you sometimes forget to bring along your medications?**	**1) Yes**<br>**2) No**
**26**	**Did you take your medications yesterday?**	**1) Yes**

		**2) No**
**27**	**When you feel like your health condition is under control, do you sometimes stop taking you medications?**	**1) Yes**<br>**2) No**
**28**	**Taking medications every day is a real inconvenience for some people. Do you ever feel hassled about sticking to your treatment plan?**	**1) Yes**<br>**2) No**
**29**	**How often do you have difficulty remembering to take all your medications?**	**4) Never/rarely**<br>**3) Occasionally**<br>**2) Sometimes**<br>**1) Usually**<br>**0) All the time**
**The Brief Illness Perception Questionnaire**		
**30**	**How much does your illness affect your life?**	**0) No affect at all**<br>**1)**<br>**2)**<br>**3)**<br>**4)**<br>**5)**<br>**6)**<br>**7)**<br>**8)**<br>**9)**<br>**10) severely affect my life**
**31**	**How long do you think your illness will continue?**	**0) A very short time**<br>**1)**<br>**2)**<br>**3)**<br>**4)**<br>**5)**<br>**6)**<br>**7)**<br>**8)**<br>**9)**<br>**10) Forever**

**32**	**How much control do you feel you have over your illness?**	**0) No control at all**<br>**1)**<br>**2)**<br>**3)**<br>**4)**<br>**5)**<br>**6)**<br>**7)**<br>**8)**<br>**9)**<br>**10) extremely amount of control**
**33**	**How much do you think your treatment can help your illness?**	**0) Not at all**<br>**1)**<br>**2)**<br>**3)**<br>**4)**<br>**5)**<br>**6)**<br>**7)**<br>**8)**<br>**9)**<br>**10) extremely helpful**
**34**	**How much do you experience symptoms from your illness?**	**0) No symptoms at all**<br>**1)**<br>**2)**<br>**3)**<br>**4)**<br>**5)**<br>**6)**<br>**7)**<br>**8)**<br>**9)**<br>**10) many sever symptoms**
**35**	**How concerned are you about your illness?**	**0) Not at all concerned**<br>**1)**<br>**2)**<br>**3)**<br>**4)**<br>**5)**<br>**6)**

		7)<br>8)<br>9)<br>10) Extremely concerned
**36**	**How well do you feel you understand your illness?**	**0) Don't understand at all**<br>**1)**<br>**2)**<br>**3)**<br>**4)**<br>**5)**<br>**6)**<br>**7)**<br>**8)**<br>**9)**<br>**10) understand very clearly**
**37**	**How much does your illness affect you emotionally? (e.g., does it make you angry, scared, upset or depressed?**	**0) Not at all affected emotionally**<br>**1)**<br>**2)**<br>**3)**<br>**4)**<br>**5)**<br>**6)**<br>**7)**<br>**8)**<br>**9)**<br>**10) Extremely affected emotionally**
**38**	**Please list in rank-order the three most important factors that you believe caused your illness. The most important causes for me- :**	**1.** ____________________________________<br>**2.**____________________________________<br>**3.**____________________________________
